# Adolescent Nutritional Patterns and Health Behaviors in Romania: A Cross-Sectional Analysis

**DOI:** 10.3390/nu17091448

**Published:** 2025-04-25

**Authors:** Carmen Elena Lupu, Alexandru Scafa-Udriște, Raluca Silvia Matei, Monica Licu, Tiberius Iustinian Stanciu, Gabriela Stanciu, Fallah Hashemi, Andreea Mihai, Sergiu Lupu, Răzvan Ene, Radu Emilian Cristache, Steluța Constanța Boroghină, Anca Coliță, Octavian Buda, Magdalena Mititelu

**Affiliations:** 1 Department of Mathematics and Informatics, Faculty of Pharmacy, “Ovidius” University of Constanta, 900001 Constanta, Romania; clupu@univ-ovidius.ro; 2 Department of Cardio-Thoracic Pathology, Faculty of Medicine, “Carol Davila” University of Medicine and Pharmacy, 050474 Bucharest, Romania; alexandru.scafa@umfcd.ro; 3 Faculty Psychology & Educational Sciences, “Ovidius” University of Constanta, 900527 Constanta, Romania; raluca.matei@365.univ-ovidius.ro; 4 Department of Ethics and Academic Integrity, Faculty of Medicine, “Carol Davila” University of Medicine and Pharmacy, 050474 Bucharest, Romania; 5 Press Office, “Ovidius” University of Constanța, 900527 Constanta, Romania; 6 Department of Chemistry and Chemical Engineering, “Ovidius” University of Constanta, 900527 Constanta, Romania; gstanciu@univ-ovidius.ro; 7 Department of Environmental Health Engineering, School of Health, Shiraz University of Medical Sciences, Shiraz 71348-14336, Iran; info.foo@gmail.com; 8 Municipal Hospital Orșova, 225200 Orșova, Romania; andreea.mihai@umfcv.ro; 9 Department of Navigation and Naval Transport, Faculty of Navigation and Naval Management, “Mircea cel Batran” Naval Academy, 900218 Constanta, Romania; sergiu.lupu@anmb.ro; 10 Clinical Department No. 14, Faculty of Medicine, “Carol Davila” University of Medicine and Pharmacy, 050474 Bucharest, Romania; razvan.ene@umfcd.ro; 11 Faculty of Medicine, Utrecht University, 3584 CS Utrecht, The Netherlands; r.e.cristache@students.uu.nl; 12 Department of Complementary Sciences, History of Medicine and Medical Culture, Faculty of Medicine, “Carol Davila” University of Medicine and Pharmacy, 050474 Bucharest, Romania; steluta.boroghina@umfcd.ro (S.C.B.); octavian.buda@umfcd.ro (O.B.); 13 Department of Pediatrics, Faculty of Medicine, “Carol Davila” University of Medicine and Pharmacy, 050474 Bucharest, Romania; anca.colita@umfcd.ro; 14 Department of Clinical Laboratory and Food Safety, Faculty of Pharmacy, “Carol Davila” University of Medicine and Pharmacy, 020956 Bucharest, Romania; magdalena.mititelu@umfcd.ro

**Keywords:** adolescent nutrition, dietary habits, gender differences, socio-demographic factors, health behaviors, nutritional interventions

## Abstract

**Background**: Adolescence is a pivotal developmental stage marked by physiological and behavioral changes that significantly influence dietary habits and long-term health outcomes. **Objectives:** This study aimed to examine the associations between socio-demographic characteristics, psychological factors, and dietary behaviors among school-aged children and adolescents in Romania. **Methods**: A cross-sectional study was conducted using a structured, self-administered questionnaire completed by 662 children and adolescents aged 6 to 18 years, enrolled in primary, middle, and high schools across urban and rural areas. The questionnaire evaluated nutritional intake, meal patterns, hydration habits, physical activity, screen time, and psychological factors such as fatigue, stress, and mood fluctuations. Body mass index (BMI) was calculated based on self-reported height and weight. **Results**: The analysis reveals significant gender differences in body mass index (BMI), weight-loss behaviors, and family structure. Female adolescents were more likely to be underweight (18.27%) compared to male adolescents (10.82%), while overweight prevalence was higher among male adolescents (18.66%) than female adolescents (11.68%) (χ^2^ = 11.457, *p* = 0.009). Dietary patterns varied significantly across age groups, with high-school-aged children demonstrating lower vegetable consumption and increased autonomy over food choices. Primary school children exhibited higher intake of carbonated beverages (OR = 0.185, *p* = 0.002 for high school; OR = 0.116, *p* < 0.001 for middle school), whereas teenagers showed a preference for structured meals and lower fast-food intake. Meat consumption frequency correlated with greater diversity in protein sources (χ^2^ = 48.77, p < 0.001), while chaotic eating behaviors were more prevalent among middle and high-school-aged children (OR = 2.291, *p* = 0.032 for middle school; OR = 2.225, *p* = 0.030 for high school). Hydration habits played an essential role in dietary quality, with school-age children consuming ≥ 3 L of water daily exhibiting healthier eating patterns (OR = 7.146, *p* = 0.001). Additionally, psychological factors such as fatigue and stress were significantly associated with unhealthy dietary choice. **Conclusions**: These findings highlight the need for targeted nutritional education, school-based interventions, and family-focused strategies to improve adolescent health behaviors.

## 1. Introduction

Adolescence is a critical period of physical and psychological development, during which nutrition and lifestyle choices play a fundamental role in mental well-being. A balanced diet rich in essential nutrients, such as omega-3 fatty acids, vitamins B and D, calcium, and antioxidants, supports brain function, emotional regulation, cognitive performance, and bone health [1,2,3]. Conversely, diets high in processed foods, sugar, and unhealthy fats have been linked to an increased risk of anxiety, depression, and mood instability [4,5]. In addition to diet, lifestyle factors such as physical activity, sleep quality, and screen time significantly impact adolescents’ psychological health. Regular exercise promotes the release of endorphins and reduces stress, while insufficient sleep and excessive screen exposure contribute to irritability, poor concentration, and emotional distress. Understanding the complex interaction between nutrition, lifestyle, and mental health is essential for developing effective strategies to promote psychological resilience and overall well-being in adolescents [6].

A healthy diet is defined as one that includes regular consumption of vegetables, fruits, whole grains, lean proteins, and adequate hydration, while minimizing intake of added sugars, saturated fats, and ultra-processed foods [1,2,3]. In contrast, chaotic eating behavior refers to irregular meal patterns, frequent meal skipping, binge eating, and absence of structured mealtimes, which are associated with poor diet quality, psychological distress, and long-term health risks [4,5,6].

Proper nutrition during childhood and adolescence is fundamental to the development of physical, cognitive, and emotional well-being. During these critical growth stages, the body requires essential nutrients, including proteins, vitamins, minerals, and healthy fats, to support the rapid growth and development of tissues, organs, and the brain. Adequate nutrition also plays a pivotal role in strengthening the immune system, ensuring optimal energy levels, and fostering healthy bone development. Moreover, a balanced diet influences behavior, mood, and academic performance, ultimately shaping long-term health outcomes. Establishing healthy eating habits early in life can reduce the risk of developing chronic conditions such as obesity, diabetes, and cardiovascular diseases, while contributing to the formation of lifelong patterns of health and wellness. Therefore, prioritizing nutrition in childhood and adolescence is key to promoting a healthier, more prosperous future [7,8].

Balanced nutrition in children and adolescents is influenced by a variety of factors, including socio-economic status, cultural practices, access to nutritious foods, and the influence of family and peers. Socio-economic constraints often limit access to fresh, nutrient-dense foods, leading to a reliance on processed, calorie-dense options that are lower in essential nutrients. Cultural and familial dietary traditions also play a significant role in shaping eating habits, which may either promote or hinder balanced nutrition depending on the emphasis placed on nutrient-rich foods [9,10,11,12]. Additionally, the increasing prevalence of sedentary behaviors, such as excessive screen time, alongside a lack of physical activity, can contribute to poor dietary choices and unhealthy weight gain. The role of schools and community environments is also critical, as these institutions often serve as primary sources of nutrition education and meal options for adolescents. Thus, achieving balanced nutrition requires addressing these multifaceted influences through education, policy interventions, and improving access to healthy foods across different settings [13,14,15].

An unbalanced diet during childhood and adolescence can have significant medium- and long-term consequences on physical, cognitive, and emotional health. In the medium term, poor dietary habits can lead to issues such as nutrient deficiencies, which may impair growth, immune function, and cognitive development. Conditions such as anemia, poor bone health, and stunted growth are common among children with inadequate nutrition [16,17]. In the long term, the cumulative effects of an unbalanced diet can increase the risk of chronic health conditions, including obesity, type 2 diabetes, cardiovascular diseases, and certain types of cancer [18,19]. Moreover, poor nutrition during these critical developmental stages can negatively affect academic performance, behavior, and mental health, contributing to lower self-esteem and increased risk of mental health disorders like depression and anxiety [20,21,22]. These long-term health implications underscore the importance of promoting balanced nutrition early in life to mitigate future health risks and support optimal development and well-being.

Recent data indicate a significant increase in the prevalence of metabolic pathologies among children and adolescents globally and within Europe. According to a study published in *The Lancet* in March 2025, projections suggest that by 2050, over half of adults and nearly a third of children and adolescents worldwide will be overweight or obese, totaling more than 3.8 billion adults and 746 million youths. This study highlights a “monumental societal failure” to address rising obesity rates, emphasizing the urgent need for effective public health interventions [23].

In the European context, the World Health Organization’s European Childhood Obesity Surveillance Initiative (COSI) provides valuable insights. Data from the fifth round of COSI, conducted between 2018 and 2020 across 33 countries, revealed that approximately 25% of children aged 6–9 years were overweight or obese. Notably, countries such as Spain reported that in 2019, about 40.6% of children in this age group were overweight, with 17.3% classified as obese [24].

Looking ahead, the World Obesity Atlas 2023 projects that childhood obesity will more than double from 2020 levels by 2035, with a 100% increase in boys to 208 million and a 125% increase in girls to 175 million. These trends are particularly concerning given the associated health risks, including type 2 diabetes, cardiovascular diseases, and certain cancers [25].

These findings underscore the critical need for comprehensive public health strategies aimed at promoting healthy diets, increasing physical activity, and implementing policies that create supportive environments for children and adolescents. Addressing the root causes of these rising obesity rates is essential to mitigating future health risks and ensuring the well-being of future generations.

Recent data on the prevalence of metabolic pathologies among children and adolescents in Romania are limited. A study conducted in Bucharest in 2015 involving 866 participants aged 6–18 years found a significant prevalence of obesity and unhealthy behaviors among Romanian youth. The age group of 6–10.9 years exhibited a higher prevalence (40.7%) of overweight compared to the 11–17.9 age group (26.6%). However, more recent national data are scarce, highlighting the need for updated research to accurately assess current trends and inform public health strategies [26].

Another Romanian study indicates a significant prevalence of overweight and obesity among children and adolescents, with variations based on measurement standards and age groups. A pooled analysis of studies conducted between 2006 and 2015 revealed that 34.7% of 8-year-old children were overweight, with 16.2% classified as obese [27].

Similarly, the World Health Organization’s Childhood Obesity Surveillance Initiative (COSI) reported that between 2018 and 2020, 8-year-old children in Romania had an overweight prevalence of 25.2% and an obesity prevalence of 10%, based on WHO standards [28]. Gender differences are evident, with boys exhibiting higher rates of overweight and obesity compared to girls. Additionally, the incidence of obesity varies across different age groups, highlighting the need for targeted interventions. These findings underscore the urgency for comprehensive public health strategies in Romania to address the growing concerns of childhood and adolescent obesity. Such strategies should focus on promoting healthy eating habits, increasing physical activity, and implementing policies aimed at creating supportive environments for youth health.

Assessing the impact of behavioral and nutritional risk factors in children is essential for understanding their long-term health outcomes, particularly regarding the development of metabolic pathologies. Early identification of unhealthy eating habits, sedentary behaviors, and other risk factors enables timely interventions to prevent the onset of conditions such as obesity, type 2 diabetes, and cardiovascular diseases. Since childhood is a critical period for physical and metabolic development, the effects of poor nutrition and lifestyle choices can have lasting consequences, influencing not only immediate health but also the trajectory of health throughout adulthood. By evaluating these risk factors, healthcare professionals can better understand the complex relationship between diet, behavior, and disease onset, leading to more targeted and effective preventive strategies. Furthermore, such assessments provide valuable insights into the broader public health landscape, informing policies and interventions aimed at reducing the prevalence of metabolic disorders among future generations.

Proper nutrition during childhood and adolescence plays a fundamental role in physical growth, cognitive development, and the prevention of chronic diseases later in life [8,9]. The recent literature highlights the increasing prevalence of unhealthy eating behaviors among young populations, characterized by low intake of fruits and vegetables, high consumption of ultra-processed foods, and irregular meal patterns [12,13]. These behaviors are influenced by a complex array of socio-demographic and psychological factors, including family structure, gender norms, urbanization, school stress, and exposure to social media [16,18,19].

Furthermore, dietary behaviors have been associated with emotional well-being, body image, and sedentary lifestyles, underscoring the need for a more integrated understanding of adolescent nutrition [6,14,17]. While many studies have focused on specific dietary components or isolated health outcomes, few have comprehensively assessed the interaction between dietary patterns, socio-demographic variables, and psychological status in a representative school-aged population [7,10].

This cross-sectional study aimed to assess the dietary habits, health behaviors, and socio-demographic characteristics of school-aged children and adolescents. By identifying critical determinants of eating behavior, this study contributes to the development of targeted, evidence-based nutritional interventions for youth.

This study analyzes the dietary habits, socio-demographic influences, and health-related behaviors of school-aged children and adolescents to identify key patterns and risk factors associated with unhealthy eating behaviors.

By assessing the impact of gender, age, family structure, and psychological factors on food choices, this study seeks to provide evidence-based recommendations for targeted nutritional interventions and educational programs aimed at promoting healthier eating habits and preventing diet-related health issues among young populations.

## 2. Materials and Methods

Data collection was conducted using a structured, self-administered questionnaire distributed across several educational institutions. The questionnaire was designed to gather comprehensive information on participants’ nutritional intake, meal patterns, psychological well-being, and lifestyle factors, including physical activity and screen time.

A total of 662 valid responses were collected from students aged between 6 and 18 years. Participants were stratified by gender, educational level (primary, middle, or high school), residence (urban or rural), family structure, and body mass index (BMI), which was calculated based on self-reported weight and height using standard WHO z-score classifications.

### 2.1. Study Design

This study employs a cross-sectional observational design to assess the dietary behaviors and nutritional preferences of school-aged children in relation to socio-demographic determinants. Data collection was conducted using validated dietary assessment questionnaires [29,30] and socio-economic surveys administered to both children and their parents. This study was conducted in multiple educational institutions across urban and rural areas to ensure a representative sample. The inclusion criterion was regular school attendance. The exclusion criteria were diagnosis of metabolic disorders affecting diet (e.g., diabetes, celiac disease) and incomplete responses in the survey.

The questionnaire included both closed-ended and multiple-choice items covering food frequency, type of meals consumed (e.g., home-cooked, fast food), hydration habits, and perceptions of health. Psychological well-being was assessed through items measuring fatigue, stress, mood fluctuations, and sleep quality. The tool was piloted for clarity and relevance prior to distribution. The Validated Food Frequency Questionnaire (FFQ) was used to assess the frequency and types of foods consumed. Dietary Diversity Scores (DDSs) were calculated based on food group consumption to assess nutritional balance.

The questionnaire was piloted for clarity and age appropriateness with a subsample of 30 school children. In addition to testing usability, internal consistency was assessed using Cronbach’s alpha. The resulting coefficient was α = 0.81, indicating good reliability. Construct and content validity were reviewed by three academic experts in adolescent nutrition and psychology.

Participants were selected using a stratified convenience sampling method, ensuring balanced representation across school levels (primary, middle, and high school) and geographic areas (urban and rural). Although not random, this stratified approach allowed demographic diversity and supported comparative subgroup analysis.

### 2.2. Statistical Analysis

Statistical analyses were conducted using IBM SPSS Statistics for Windows, version 30.0 (IBM Corp., Armonk, NY, USA). Descriptive statistics were used to characterize the sample. Chi-square tests assessed associations between categorical variables. Multinomial logistic regression was applied to examine the influence of socio-demographic and behavioral factors on educational level and dietary outcomes. Odds ratios (ORs) and 95% confidence intervals (CIs) were calculated, with the significance level set at *p* < 0.05.

All data were collected anonymously, and participation was voluntary. Parental consent was obtained for participants under 18 years of age. This study was conducted in accordance with the Declaration of Helsinki and approved by the Ethics Commission of the Carol Davila University of Medicine and Pharmacy in Bucharest Romania (no. 4201/16.02.2024).

## 3. Results

Following the dissemination of the questionnaire, 662 responses were collected (Table 1), with 59.52% of respondents being female adolescents and 40.48% male adolescents. The respondents were categorized based on various socio-demographic and anthropometric factors, including BMI, residence area, education level, family structure, weight-loss behaviors, and family history of excessive weight.

A significant association was observed between BMI and gender (χ^2^ = 11.457, *p* = 0.009). Most respondents fell within the normal BMI range (66.61%), with minimal gender differences (66.75% females vs. 66.42% males). However, notable discrepancies were present in the underweight and overweight categories.

Educational level distribution was significantly associated with gender (χ^2^ = 32.425, *p* < 0.001). A higher percentage of female adolescents were high school students (45.94%) compared to male adolescents (28.36%), whereas a greater proportion of male adolescents were in primary school (42.91%) compared to female adolescents (23.35%).

Weight-loss behaviors demonstrated a highly significant association with gender (χ^2^ = 21.300, *p* < 0.001).

Family structure also showed significant gender differences (χ^2^ = 13.727, *p* = 0.008). Male adolescents were more likely to be only children (35.82%) compared to female adolescents (25.63%), while female adolescents were more likely to have three siblings (5.08%) compared to males (1.12%). Family structure may play a role in shaping health behaviors, as children from larger families may have different social dynamics, dietary habits, and physical activity patterns compared to only children.

No significant gender differences were observed regarding the presence of excessive weight in the family (χ^2^ = 2.947, *p* = 0.400). Most respondents reported no family history of excessive weight (73.57%), with slight variations between female adolescents (71.83%) and male adolescents (76.12%). This suggests that familial weight issues may not differ substantially between genders within this cohort.

### 3.1. The Influence of Socio-Demographic Factors on Adolescent Nutritional Patterns

Table 2 presents the results of the multinomial logistic regression analysis, evaluating the association between educational level (high school and middle school vs. primary school) and socio-demographic factors, BMI, and dietary habits. Odds ratios (ORs) and 95% confidence intervals (CIs) are calculated to identify significant trends in adolescents’ dietary behaviors. High school students who consume vegetables two or three times per week have significantly higher odds of being in high school compared to primary school students (OR = 1.603, *p* = 0.082), but this effect weakens in middle school students (OR = 1.290, *p* = 0.373). These findings suggest that higher vegetable consumption may correlate with higher educational attainment, though the relationship is not strongly defined. Adolescents who consume fast food less frequently (less than once a week) are more likely to be in high school. The odds ratio for those eating fast food 2–3 times a week is significantly lower in high school adolescents (OR = 0.121, *p* = 0.018). This suggests that reducing fast-food consumption could have a positive association with academic outcomes, as adolescents in high school tend to have healthier eating habits regarding fast food.

### 3.2. Dietary Habits Among Adolescents

**Figure 1:**
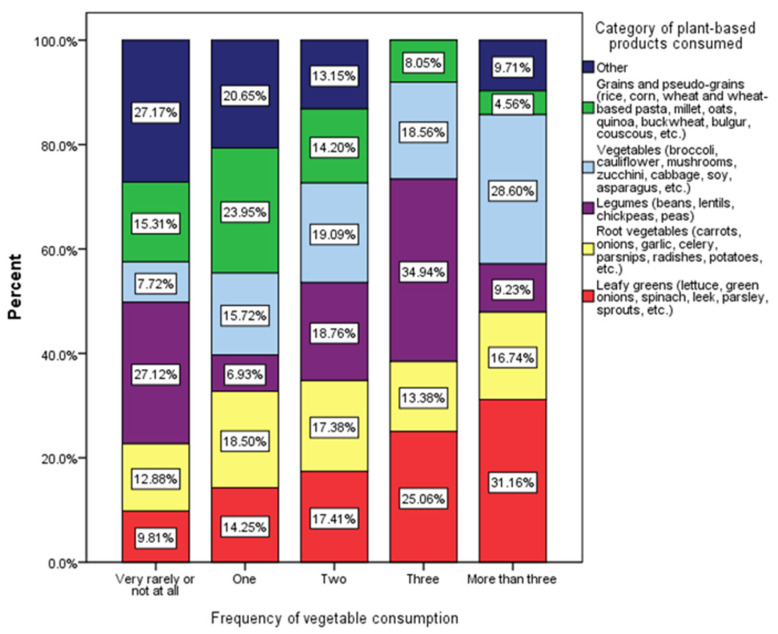
Distribution of plant-based food categories by frequency of vegetable consumption among respondents (χ^2^ = 55.07, *p* < 0.001).

Figure 1 illustrates how the frequency of vegetable consumption correlates with the diversity of plant-based food categories consumed. The categories examined include grains and pseudo-grains (e.g., rice, corn, wheat, quinoa), various vegetables (e.g., broccoli, mushrooms, zucchini), legumes (e.g., beans, lentils, soy), root vegetables (e.g., carrots, beets, potatoes), leafy greens (e.g., spinach, leek, parsley), and a miscellaneous category labeled as “other plant-based foods”. The Chi-square test results (χ^2^ = 55.07, *p* < 0.001) indicate a statistically significant association between the frequency of vegetable consumption and the diversity of plant-based products in respondents’ diets.

**Figure 2:**
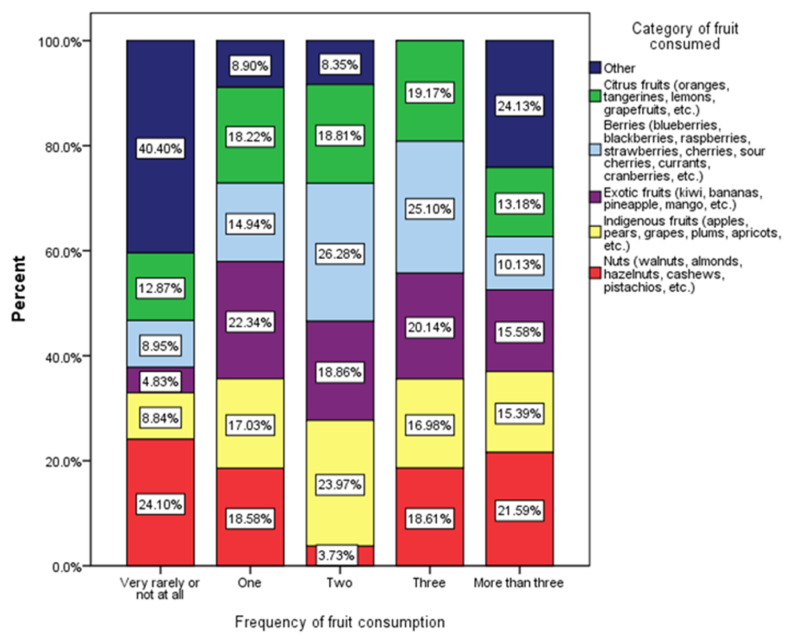
Distribution of fruit categories by frequency of fruit consumption among respondents (χ^2^ = 51.08, *p* < 0.001).

Figure 2 presents the distribution of fruit categories consumed by respondents based on the frequency of their fruit consumption. The fruit categories analyzed include citrus fruits (e.g., oranges, lemons, grapefruits), berries (e.g., blueberries, strawberries, raspberries), exotic fruits (e.g., kiwi, bananas, pineapples), indigenous fruits (e.g., apples, pears, grapes), and nuts (e.g., walnuts, almonds, hazelnuts). The “other” category encompasses less common fruits or unspecified types.

The Chi-square test reveals a statistically significant association between the frequency of fruit consumption and the diversity of fruit categories consumed (χ^2^ = 51.08, *p* < 0.001). This suggests that individuals who consume fruits more frequently incorporate a broader variety of fruit types into their diet.

**Figure 3:**
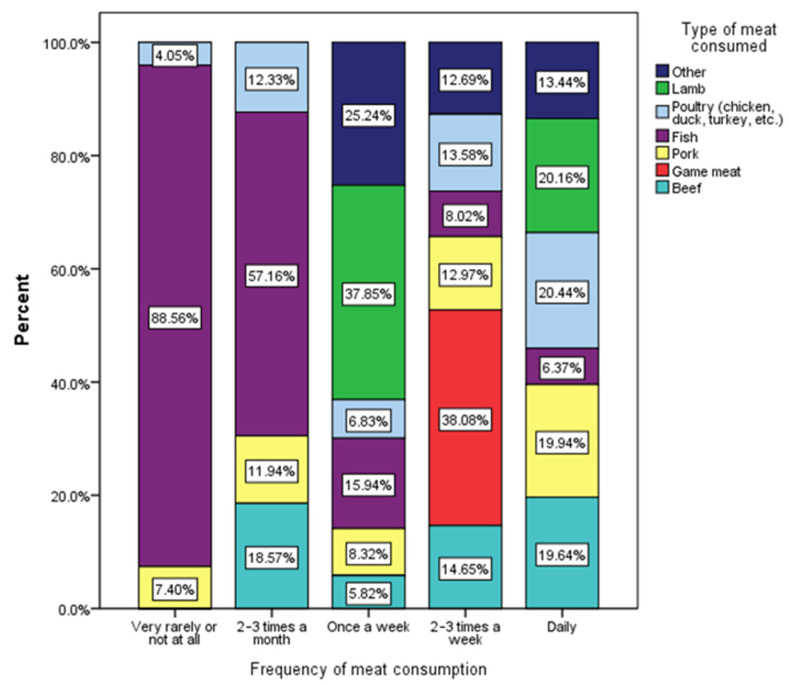
Distribution of meat types by frequency of meat consumption among respondents (χ^2^ = 48.77, *p* < 0.001).

Figure 3 illustrates the distribution of various meat types consumed by respondents in relation to the frequency of their meat consumption. The analyzed meat categories include poultry (such as chicken, duck, and turkey), fish, pork, game meat, beef, lamb, and a miscellaneous category labeled as “other”.

The Chi-square test result (χ^2^ = 48.77, *p* < 0.001) indicates a statistically significant association between the frequency of meat consumption and the types of meat incorporated into the diet. This suggests that individuals who consume meat more frequently tend to diversify their meat choices compared to those who consume it infrequently.

### 3.3. The Impact of Dietary Patterns and Lifestyle on Adolescent Well-Being

The analysis of dietary habits among adolescents, particularly as they transition from primary to middle and high school, reveals significant changes in food consumption patterns that can impact their health outcomes. The data presented in Table 3 highlight various factors influencing dietary choices, including the type of food consumed, cooking methods, and the overall assessment of eating behaviors.

Adolescence is a stage characterized by psychological and behavioral shifts that in-fluence overall well-being. To explore how these factors shape health-related behaviors, a multinomial logistic regression analysis was performed. Table 4 displays the findings, highlighting the relationships between stress, fatigue, mental health symptoms, and lifestyle habits across different educational levels. For high school students, there is a significant negative association with daily exercise for less than an hour (OR = 0.231, *p* = 0.008), indicating they are less likely to engage in this level of physical activity compared to primary school students.

For middle school, there were no significant associations with physical activity levels across the different exercise categories compared to primary school.

This suggests that high school students might be more sedentary or engage in shorter periods of physical activity than primary school students.

## 4. Discussion

Proper nutrition during childhood and adolescence plays an essential role in shaping long-term health outcomes, influencing growth, cognitive development, and the prevention of chronic diseases. However, modern dietary habits are increasingly shifting toward processed and nutrient-poor foods, driven by socio-economic factors, cultural influences, and food availability. Understanding the dietary patterns of school-aged children is essential for developing targeted interventions that promote balanced nutrition. This study explores the complex interplay between socio-demographic factors and dietary behaviors, aiming to identify key determinants that influence food choices among children. By addressing these factors, the research seeks to provide valuable insights for policymakers, educators, and healthcare professionals striving to enhance nutritional education and food accessibility in school environments.

Female adolescents were more likely to be underweight (18.27%) compared to male adolescents (10.82%), possibly reflecting societal pressures on girls to maintain a slimmer physique (Table 1). Conversely, overweight prevalence was higher among male adolescents (18.66%) than female adolescents (11.68%) [31].

Female adolescents reported engaging in weight-loss diets more frequently, with 8.38% indicating they do so very often and 26.65% sometimes, compared to only 3.73% and 15.30% of male adolescents, respectively. Male adolescents were more likely to report not engaging in weight-loss behaviors at all (69.40% vs. 53.55% for female adolescents). These findings are consistent with previous studies that highlight the prevalence of dieting behaviors among adolescent girls, often driven by body image concerns and societal expectations [32].

As concerns the dietary habits of adolescents, one significant trend is the association between the frequency of vegetable consumption and educational level. High school students who rarely or never consume vegetables have a significantly increased risk of unhealthy dietary behaviors (OR = 0.418, *p* = 0.023) compared to primary school students (Table 2). Similarly, middle school students show a tendency toward reduced vegetable intake, though this is not statistically significant. This reduction may reflect the growing independence of older students and their increased exposure to less nutritious food options outside the home [33]. Studies have shown that adolescents often replace vegetables with energy-dense, nutrient-poor foods as they gain more autonomy over their dietary choices [34].

The consumption of carbonated or sweetened drinks also highlights critical differences. Both high school and middle school students show a substantially lower likelihood of frequent consumption compared to primary school students (OR = 0.185, *p* = 0.002 for high school; OR = 0.116, *p* < 0.001 for middle school). This suggests that younger students are more exposed to these sugary beverages, possibly due to parental influence or school policies that limit access to such drinks in older age groups. However, this trend may vary depending on socio-economic factors and regional dietary norms [35]. For example, adolescents in rural areas may have less access to sugary drinks due to limited availability, while urban adolescents may face greater exposure to marketing and convenience stores.

The analysis of meat consumption patterns (Table 2) reveals that middle school students who consume meat very rarely or not at all have a significantly increased risk of unhealthy dietary behaviors (OR = 6.578, *p* = 0.039). This could indicate a shift toward imbalanced diets lacking essential proteins and nutrients, possibly influenced by dietary fads, misinformation, or the adoption of plant-based diets without proper nutritional guidance [36]. The lack of meat in the diet may lead to deficiencies in iron, vitamin B12, and other essential nutrients, particularly if not replaced with adequate plant-based protein sources.

Interestingly, the frequency of fast-food consumption did not show a strong association with unhealthy dietary habits among high school students but indicated a risk among middle school students. This aligns with findings from global studies on the impact of fast-food marketing targeting younger demographics [37]. Middle school students may be more susceptible to fast-food advertising and peer influence, leading to higher consumption rates. However, high school students may have greater awareness of the health risks associated with fast food or may have developed more stable dietary habits.

The frequency of water consumption also highlights a critical shift. Middle school students who consume 3 L of water daily have a significantly higher likelihood of healthy dietary habits (OR = 7.146, *p* = 0.001), suggesting that hydration plays an essential role in overall health and dietary behavior (Table 2). This aligns with research indicating the importance of adequate hydration in maintaining cognitive and physical performance in adolescents [38]. Proper hydration is also associated with better appetite regulation and reduced consumption of sugary beverages.

The results indicate a significant association between the frequency of vegetable consumption and the diversity of plant-based products in respondents’ diets (Figure 1). This suggests that individuals who consume vegetables more frequently tend to have a more varied and nutritionally balanced diet, incorporating a wider range of plant-based food categories. This finding is consistent with previous research indicating that higher vegetable intake is associated with improved diet quality and better health outcomes, particularly in adolescents [39]. Respondents who reported very rare or no vegetable consumption predominantly relied on grains and pseudo-grains, which constituted 27.71% of their intake, while their consumption of vegetables and leafy greens remained minimal at 7.72% and 9.81%, respectively. This dietary pattern suggests a preference for processed or grain-based foods, which may contribute to less balanced nutritional profiles. In contrast, participants consuming one or two categories of vegetables exhibited a more diverse plant-based intake, with higher proportions of root vegetables and general vegetables, indicating a transitional stage towards healthier eating patterns [40]. A notable dietary shift occurs among respondents consuming three or more vegetable categories. In these groups, vegetable intake significantly increases, with vegetables comprising 18.56% for those consuming three categories and 28.60% for those consuming more than three.

The analysis of fruit consumption patterns among respondents reveals significant in-sights into dietary diversity and health-related behaviors (Figure 2). Individuals who reported consuming fruits very rarely or not at all (24.10%) predominantly relied on items categorized as “other” (40.40%). Their intake of indigenous fruits (8.84%) and citrus fruits (8.65%) was notably lower compared to those with higher fruit consumption. This pattern suggests a limited variety of fruits and possibly a higher reliance on processed or non-fresh fruit options, which may negatively impact their overall nutritional intake [41]. In the moderate fruit consumption category, participants consuming one (18.56%) or two (23.97%) types of fruits demonstrated an increased intake of diverse fruit categories. For instance, those consuming two types of fruits had higher proportions of berries (26.28%) and citrus fruits (18.86%). This indicates a transition towards more balanced fruit consumption patterns, which is essential for achieving recommended dietary guidelines. A substantial shift is evident in respondents who consume three (18.61%) or more than three (19.51%) types of fruits. These groups showed a marked increase in the consumption of indigenous fruits (16.98% for three types; 15.58% for more than three) and nuts (18.61% for three types; 15.59% for more than three). Conversely, the proportion of “other” fruits declined to 19.17% and 24.13%, indicating a stronger focus on nutrient-rich, commonly consumed fruits. This shift aligns with findings that suggest higher fruit and nut consumption is associated with improved overall diet quality and better health outcomes, including reduced risk of chronic diseases and enhanced immune function [42,43]. The data reveal that increased frequency of fruit consumption is associated with greater dietary diversity and a shift towards nutrient-rich fruit categories, such as berries, nuts, and indigenous fruits. This aligns with the existing literature indicating that higher fruit consumption contributes to better overall diet quality and improved health outcomes, including reduced risk of chronic diseases and enhanced immune function [42,44]. The promotion of indigenous fruits and nuts is particularly noteworthy, as these foods are often rich in essential nutrients and can play a significant role in addressing dietary deficiencies in various populations [43,44]. Furthermore, the integration of indigenous foods into diets has been shown to enhance nutritional security and improve health outcomes, particularly in communities that traditionally rely on these foods [43].

Respondents who reported consuming meat very rarely or not at all predominantly fall into the “other” category, with 88.56% likely indicating vegetarian or plant-based preferences (Figure 3). Only 7.40% of these individuals consume poultry, with negligible consumption of other meat types. This suggests a minimal reliance on meat, potentially driven by dietary restrictions, health considerations, or personal preferences. In Romania, the adoption of vegetarian or plant-based diets has been growing, particularly among younger populations, influenced by global health trends and environmental concerns [45]. However, without proper nutritional guidance, such diets may lead to deficiencies in essential nutrients like iron, vitamin B12, and protein [46].

In contrast, respondents who consume meat 2–3 times a month exhibit consumption of a broader variety of meat types. Poultry accounts for 18.57% of their consumption, while fish constitutes 15.44%. Pork represents 11.94%, indicating a gradual incorporation of diverse meats into their diet as consumption frequency increases.

A notable shift is observed among respondents who consume meat once a week or more frequently. Those who eat meat once a week show higher consumption of poultry (37.85%) and fish (15.44%), with a moderate intake of pork (6.83%). As meat consumption frequency increases to 2–3 times a week, pork becomes the dominant choice, representing 38.08% of meat intake, followed by poultry (12.97%) and beef (8.32%). This group also shows a notable increase in game meat consumption (13.58%), suggesting a preference for richer, more diverse meat options.

Respondents who consume meat daily exhibit the greatest diversity in meat choices. Poultry remains significant at 20.16%, while beef (19.64%), pork (20.44%), and fish (6.37%) also feature prominently. Game meat and lamb are more frequently consumed by daily meat eaters, at 13.44% each, indicating a well-rounded, varied meat diet among this group. For example, red meats like beef and lamb are rich in iron and zinc, while poultry and fish provide lean protein and omega-3 fatty acids.

The data demonstrate a clear trend linking increased meat consumption frequency with greater diversity in meat types. Individuals who consume meat infrequently tend to rely predominantly on poultry or avoid meat altogether, whereas frequent meat consumers incorporate a wide variety of meat types, including pork, beef, fish, and game meat.

Primary school students generally exhibit healthier and more structured eating patterns compared to their older counterparts. This is likely due to the strong influence of parental supervision, school meal programs, and the absence of significant exposure to external food environments. In contrast, as children transition to middle and high school, their dietary habits tend to become less structured and more influenced by external factors such as peer pressure, academic stress, and increased independence.

One of the most notable trends is the shift toward chaotic and excessive eating among middle and high school students (Table 3). Compared to primary school students, both middle and high school students are significantly more likely to report chaotic eating patterns (OR = 2.291, *p* = 0.032 for middle school; OR = 2.225, *p* = 0.030 for high school). This aligns with global studies showing that adolescents often skip meals, snack irregularly, and consume energy-dense, nutrient-poor foods [47]. This trend is exacerbated by the increasing availability of fast food and the decline in traditional family meals, which are more common among primary school students [48].

Another key finding is the growing consumption of fast food and processed products among middle and high school students. While primary school students are more likely to consume home-cooked meals or meals provided by school canteens, older students show a tendency toward fast-food consumption (OR = 3.712, *p* = 0.166 for middle school). This reflects a broader global trend where adolescents are increasingly exposed to fast-food outlets and marketing campaigns targeting their age group [13]. Urbanization and the proliferation of fast-food chains have contributed to this shift, particularly in urban areas [49].

The role of family meals also diminishes as children grow older. Both middle and high school students are less likely to eat meals with their families compared to primary school students (OR = 0.587, *p* = 0.029 for middle school; OR = 0.438, *p* < 0.001 for high school). Family meals are a cornerstone of healthy eating habits, providing an opportunity for parents to model balanced diets and monitor their children’s food intake. The decline in family meals among older students may contribute to the adoption of less healthy eating behaviors, such as increased consumption of snacks and fast food [50].

Receptivity to new foods also decreases as children transition from primary school to adolescence. Both middle and high school students are less likely to try new foods compared to primary school students (OR = 0.370, *p* < 0.001 for middle school; OR = 0.200, *p* < 0.001 for high school). This may reflect a natural tendency toward food neophobia during adolescence, as well as the influence of peer preferences and the desire to conform to social norms.

The shift from structured, family-centered eating patterns in primary school to chaotic, fast-food-oriented diets in middle and high school has significant implications for adolescent health. Chaotic eating and excessive consumption of fast food are associated with an increased risk of obesity, cardiovascular diseases, and other chronic conditions [51]. Additionally, the decline in family meals and reduced receptivity to new foods may limit adolescents’ exposure to essential nutrients, further compromising their long-term health.

Recent studies have highlighted the need for targeted interventions to promote healthy eating habits among adolescents [45], including strengthening nutritional education, regulating the availability of unhealthy foods in schools, and encouraging family meals.

Based on the multinomial logistic regression analysis of dietary patterns and eating behaviors associated with educational level (high school and middle school vs. primary school), the findings reveal several important insights that reflect the complex relationship between educational level and food consumption behaviors. High school students are significantly less likely to consume home-cooked meals compared to primary school students, which may reflect a trend towards increased reliance on fast foods and restaurant-cooked meals as students grow older. This shift could be driven by factors such as greater autonomy, changing lifestyles, and a shift towards convenience foods as adolescents navigate academic and social demands. The consumption of oven-cooked foods is notably lower among middle school students compared to primary school students, suggesting a potential trend toward less healthy cooking methods. Although the difference is not marked in all cooking methods, these findings may point to broader shifts in food preparation styles as children grow older and have greater exposure to processed and fast foods. There is no significant difference between high school and middle school students for many food types, including vegetables, cereals, pasta, and fish, but high school students tend to be less receptive to new foods compared to their primary school counterparts. This reluctance towards trying new foods may be linked to established preferences and a resistance to unfamiliar tastes or textures, which could have implications for their long-term health outcomes. Despite the lack of significant differences for some categories, fast-food products (such as burgers, shawarma, and fries) show a tendency to be consumed more frequently among high school students. This highlights the growing influence of the fast-food industry and peer influence in adolescence, contributing to less healthy dietary habits. Both high school and middle school students are more likely to report chaotic, excessive eating patterns compared to primary school students. This finding emphasizes the importance of addressing disordered eating behaviors early on, particularly as children transition into adolescence. Hormonal changes specific to adolescence significantly influence eating behaviors through complex mechanisms, in which leptin and ghrelin regulate hunger and satiety, insulin influences carbohydrate metabolism, and cortisol, the stress hormone, can stimulate an appetite for foods rich in sugar and fat; at the same time, fluctuations in estrogen and progesterone in adolescent girls, as well as increased testosterone levels in adolescent boys, can determine specific food preferences and changes in energy metabolism, also being influenced by circadian rhythm and sleep quality. Excessive eating habits at these stages can contribute to weight gain and poor nutritional health in the long run.

Interestingly, middle school students are more likely than primary school students to monitor their weight, suggesting an increased awareness of body image and health at a younger age. This could reflect growing societal pressures related to appearance and health that influence dietary choices. School stress and anxiety profoundly influence adolescents’ eating behaviors, as elevated cortisol levels can stimulate appetite for high-calorie, sugary foods, while academic pressure, heavy homework, and exams can promote either compulsive eating as a stress management mechanism or food restriction due to decreased appetite or excessive weight concerns, with these effects exacerbated by a lack of sleep and an unbalanced daily routine.

Social media and peer influence play a key role in shaping adolescents’ eating behaviors, with the promotion of unrealistic body standards and extreme diets by influencers and celebrities contributing to the adoption of unhealthy eating habits, increased body image concerns, and, in some cases, the development of eating disorders, such as anorexia or bulimia, especially in the context of constant exposure to idealized content.

However, the relationship between weight monitoring and actual healthy eating behaviors remains complex and warrants further exploration. High school students tend to eat less frequently with family compared to primary school students. This trend suggests a shift towards more independent meal habits as adolescents seek autonomy in their dietary choices. The reduced family involvement during meals may have implications for social and emotional eating habits, potentially leading to unhealthy eating behaviors if not addressed. The family is a determining factor in the development of adolescents’ eating habits, with regular family meals, a balanced parenting style, and emotional support contributing to the formation of healthy eating behavior, while a lack of parental involvement or a tense family environment can favor chaotic eating or excessive consumption of unhealthy foods, and well-structured educational interventions in schools can play an essential role in preventing unbalanced eating behaviors by promoting a balanced diet and stress management strategies.

Both high school and middle school students exhibit significant resistance to accepting new foods, with high school students being especially reluctant. This could be attributed to the development of more ingrained food preferences and a desire for comfort and familiarity, which may hinder the adoption of healthier food choices. The findings regarding eating pace and multitasking during meals show that while high school students are not significantly different from middle school students in terms of eating speed or doing other activities while eating, these behaviors are common across age groups. Such habits may contribute to overeating or poor digestion, signaling a need for more mindful eating interventions in school settings.

Fatigue is a significant issue among middle and high school students, with both groups reporting higher levels compared to primary school students (Table 4). High school students are particularly affected, with those experiencing frequent fatigue showing a significantly higher risk of unhealthy behaviors (OR = 2.330, *p* = 0.013). Alarmingly, students who are almost always fatigued exhibit an extremely high risk (OR = 14.592, *p* < 0.001). This aligns with recent research indicating that academic pressure, poor sleep hygiene, and increased screen time contribute to chronic fatigue among adolescents [52]. Similarly, stress levels escalate as students advance through the educational system. High school students frequently report stress (OR = 2.574, *p* = 0.011), which can be attributed to academic demands, social pressures, and the transition to adulthood. This trend is consistent with findings from the World Health Organization, which highlights stress as a critical issue affecting adolescent mental health [52].

Conflictual relationships with peers become more prevalent in older students. High school students who report conflicts with peers have a significantly higher risk of adverse health outcomes (OR = 2.752, *p* = 0.025), with middle school students showing an even higher risk (OR = 3.428, *p* = 0.006). These findings suggest that peer relationships play a pivotal role in adolescents’ psychological well-being. Peer conflicts are strongly associated with anxiety, depression, and poor academic performance, as supported by recent studies [53]. Mental health concerns, particularly depressive states, also show a marked increase in high school students. Those frequently experiencing depressive symptoms have a significantly reduced likelihood of maintaining healthy behaviors (OR = 0.121, *p* = 0.005), and those almost always in depressive states exhibit similar risks (OR = 0.160, *p* = 0.045). This trend highlights the urgent need for mental health interventions in schools, echoing global calls for increased mental health resources for adolescents [54].

Fatigue and irritability are closely linked, with high school students showing higher prevalence rates. Frequent fatigue (OR = 2.330, *p* = 0.013) and irritability contribute to un-healthy lifestyle choices, as adolescents may resort to comfort foods, skip meals, or engage in irregular eating patterns. This is supported by the literature linking psychological stress and fatigue to disordered eating behaviors in adolescents [55]. Interestingly, immune system perceptions also differ across educational levels. High school students who believe they have a weakened immune system are nearly twice as likely to report unhealthy behaviors (OR = 1.999, *p* = 0.092).

Physical activity levels decline as students progress through school, with high school students who engage in daily physical activity for less than an hour showing a significantly higher likelihood of healthy behaviors (OR = 0.231, *p* = 0.008). This highlights the importance of regular physical activity in promoting overall health. Conversely, excessive screen time is associated with unhealthy behaviors. Students who spend less time in front of screens are more likely to maintain healthy dietary habits (OR = 0.195, *p* = 0.001 for middle school; OR = 0.245, *p* = 0.004 for high school). This aligns with studies linking excessive screen time to sedentary behavior and poor dietary choices [56].

For both high school and middle school students, there is a negative association with the amount of time spent in front of screens compared to primary school students (Table 4). Specifically,

- high school and middle school students are less likely to spend more than 1 h, 2–3 h, or 4–5 h daily in front of screens than primary school students;
- in both groups, students who spend a few hours 2–3 times a week in front of screens also show a significant negative association, indicating they are less likely to engage in this behavior compared to primary school students.

These findings suggest that primary school students tend to spend more time in front of screens across various categories compared to high school and middle school students.

The multinomial logistic regression analysis underscores that socio-demographic factors such as residence area and dietary habits, particularly related to the consumption of vegetables, fruits, sweetened drinks, fast food, and water, significantly influence the educational attainment of adolescents. Rural adolescents tend to be over-represented in middle school, while dietary habits, such as regular vegetable consumption, reduced intake of fast food, and increased water consumption, are associated with higher odds of being in higher educational levels (high school). These findings suggest the need for interventions that promote healthier eating habits, particularly focusing on improving the availability and consumption of nutritious foods and beverages to enhance the educational outcomes of adolescents. The results also indicate a significant evolution in dietary behaviors and eating patterns as students transition from primary school to middle and high school. While there are some commonalities, such as a tendency toward chaotic eating and reluctance to try new foods, there are also critical shifts, such as decreased consumption of home-cooked meals and increased consumption of fast food, particularly in high school students. These changes highlight the need for targeted interventions to promote healthy eating habits, mindful consumption, and family engagement during mealtimes during adolescence, a critical period for establishing long-term healthy behaviors. Moreover, the resistance to new foods and chaotic eating patterns suggest that educational programs focusing on healthy eating, cooking skills, and family meal practices could be important in mitigating the rise in unhealthy dietary trends among adolescents.

The results of this study highlight that female adolescents are more likely to report dieting behaviors, which aligns with previous findings indicating that adolescent girls frequently engage in weight control practices, often driven by body dissatisfaction and societal pressures [32,45]. Numerous studies have documented that girls are more likely than boys to perceive themselves as overweight, regardless of actual weight status, and to adopt restrictive eating patterns in response [32].

These behavioral patterns can be interpreted through the lens of established psychological frameworks. According to the Health Belief Model (HBM), adolescents are more likely to engage in preventive behaviors such as dieting when they perceive themselves at risk of social rejection or body-related criticism and when they believe these behaviors will bring tangible benefits (e.g., social acceptance, improved self-esteem) [54].

Furthermore, Social Cognitive Theory (SCT) emphasizes the impact of observational learning and social reinforcement. Adolescents frequently mimic behaviors modeled by peers or online influencers, particularly when these behaviors receive positive attention, which reinforces the adoption of specific eating styles or body ideals [54].

Social media platforms are now recognized as central influences in the development of disordered eating behaviors among adolescents. A recent study [57] demonstrates how exposure to curated content promoting thin ideals and unrealistic body standards contributes to body dissatisfaction and restrictive dieting. Adolescents are particularly vulnerable due to ongoing identity formation and increased sensitivity to peer validation [57].

In our study, the higher prevalence of weight monitoring and restrictive eating among girls may be partially explained by this constant exposure to social media messages reinforcing thinness and “clean eating” ideals, which normalize unhealthy patterns. The psychosocial mechanisms identified—such as appearance comparison, internalization of thin ideals, and validation seeking—are also consistent with prior findings from WHO reports on adolescent mental health and social media use [52,57].

Our findings support the development of multi-level, evidence-based interventions. At the school level, nutrition education should be expanded to include media literacy, addressing the impact of social platforms on food choices and body image. Family-based strategies—such as regular shared meals and parent-led food modeling—remain protective factors against disordered eating [50].

At a policy level, authorities should consider regulating online marketing of unhealthy foods targeting minors, while improving access to healthy options in school canteens. These approaches are aligned with WHO recommendations and supported by evidence indicating that comprehensive strategies yield better health outcomes in adolescents [52].

One of the major strengths of this study lies in its large and diverse sample, which allowed for a comprehensive analysis across educational levels, gender, and socio-demographic backgrounds. By including children and adolescents from primary, middle, and high school, this study captured developmental and behavioral transitions in dietary habits. Another strength is the multidimensional approach, incorporating not only dietary behaviors but also psychological well-being, family structure, perceived stress, physical activity, and screen time. Furthermore, the use of validated instruments such as the Food Frequency Questionnaire (FFQ) and the Dietary Diversity Score (DDS) enhances the reliability of dietary assessment. This integrated model provides a nuanced understanding of adolescent nutrition. The inclusion of multinomial logistic regression also allowed for the identification of independent predictors, offering a robust statistical basis for evidence-based recommendations.

### Limitations of This Study

This study has several limitations that should be considered when interpreting the findings. First, the reliance on self-reported dietary data may introduce recall bias and social desirability bias, potentially affecting the accuracy of responses. Second, the cross-sectional design of this study limits the ability to establish causal relationships between socio-demographic factors and dietary behaviors. Longitudinal studies would be necessary to examine changes in eating habits over time and their long-term health implications.

Additionally, while this study considers key socio-demographic and psychological variables, other potential influences such as cultural differences, economic constraints, and peer influence on dietary choices are not fully explored. The sample may also not be entirely representative of all school-aged children, particularly those from rural or marginalized communities with different food access conditions. Lastly, this study does not account for biochemical markers of nutritional status, which could provide more objective insights into the impact of dietary patterns on health outcomes. Future research should address these limitations by incorporating a broader representation of Romanian school-aged children and adolescents from various geographic, socio-economic, and ethnic backgrounds, as well as longitudinal approaches and biochemical assessments to strengthen the validity and applicability of the findings.

## 5. Conclusions

This study highlights the significant influence of socio-demographic factors on the dietary behaviors of school-aged children, emphasizing the need for targeted nutritional interventions to promote healthier eating habits. The findings suggest that parental education, economic status, and urban versus rural living environments play crucial roles in shaping children’s food choices and nutritional intake. Understanding these determinants is essential for developing effective policies and educational programs that encourage balanced diets and long-term health benefits.

Moreover, this study underscores the importance of integrating tailored nutritional guidance into school curricula and public health initiatives, ensuring that children from diverse backgrounds have access to appropriate dietary resources. While acknowledging its limitations, this research provides a foundation for future longitudinal studies and interventions aimed at bridging nutritional gaps and mitigating diet-related health risks in young populations. To support healthier eating habits among children and adolescents, future efforts should prioritize the integration of clear and practical nutrition education in schools, adapted to the age and level of understanding of school-aged children. Beyond formal lessons, informal programs such as workshops, interactive activities, and family-based discussions can play a crucial role.

## Figures and Tables

**Table 1:** Socio-demographic and anthropometric characteristics of respondents with z-scores.

	Total Population		Female Adolescents (A)		Male Adolescents (B)	
	n	%	n	%	n	%
	662	100	394	59.52	268	48.48
Body mass index (BMI) (χ^2^ = 11.457, *p* = 0.009)						
Normal	441	66.61	263	66.75	178	66.42
Obese	24	3.63	13	3.30	11	4.10
Overweight	96	14.50	46	11.68	50	18.66 ^A^
Underweight	101	15.26	72	18.27 ^B^	29	10.82
Residence area (χ^2^ = 0.006, *p* = 0.937)						
Urban area	441	66.62	262	66.50	179	66.79
Rural area	221	33.38	132	33.50	89	33.21
Level of education (χ^2^ = 32.425, *p* < 0.001)						
High school	257	38.82	181	45.94 ^B^	76	28.36
Middle school	198	29.91	121	30.71	77	28.73
General/primary school	207	31.27	92	23.35	115	42.91 ^A^
Siblings (χ^2^ = 13.727, *p* = 0.008)						
Only child	197	29.76	101	25.63	96	35.82 ^A^
One sibling	355	53.63	219	55.58	136	50.75
Two siblings	67	10.12	42	10.66	25	9.33
Three siblings	23	3.47	20	5.08 ^A^	3	1.12
More than three siblings	20	3.02	12	3.00	8	3.00
Weight-loss diet (χ^2^ = 21.300, *p* < 0.001)						
Yes, very often	43	6.5	33	8.38	10	3.73
Yes, sometimes	146	22.05	105	26.65 ^B^	41	15.30
Very rarely	76	11.48	45	11.42	31	11.57
Not at all	397	59.97	211	53.55	186	69.40 ^A^
Excessive weight in family (χ^2^ = 2.947, *p* = 0.400)						
Yes, both parents	44	6.64	28	7.11	16	5.97
Yes, one parent	122	18.43	79	20.05	43	16.04
Yes, the whole family	9	1.36	4	1.02	5	1.87
Not at all	487	73.57	283	71.83	204	76.12

Chi-square statistic significant at the 0.05 level. ^A,B^ indicate statistically significant differences between the compared categories.

**Table 2:** Multinomial logistic regression analysis of educational attainment (high school and middle school vs. primary school) in relation to socio-demographic factors, body mass index (BMI), and dietary habits.

Independent Variables	High School			Middle School		
	OR	95% CI	*p*-Value	OR	95% CI	*p*-Value
Gender						
Male adolescents	1			1		
Female adolescents	0.619	(0.363–1.055)	0.078	0.822	(0.538–1.256)	0.365
Residence area						
Urban area	1			1		
Rural area	0.889	(0.601–1.314)	0.554	1.849	(1.037–2.922)	0.008
Body mass index (BMI)						
Underweight (<18.5)	1			1		
Normal (18.5–24.9)	0.715	(0.389–1.315)	0.281	1.211	(0.630–2.327)	0.566
Overweight (25–29.9)	0.865	(0.383–1.954)	0.728	1.570	(0.658–3.747)	0.309
Obese (≥30)	0.996	(0.307–3.233)	0.995	1.197	(0.296–4.847)	0.801
Frequency of vegetable consumption						
Very rarely or not at all	0.418	(0.198–0.888)	0.023	0.635	(0.295–1.366)	0.245
One	1			1		
Two	1.603	(0.942–2.727)	0.082	1.290	(0.737–2.257)	0.373
Three	2.447	(0.980–6.112)	0.055	1.481	(0.536–4.095)	0.449
More than three	1.865	(0.626–5.558)	0.263	1.225	(0.406–3.695)	0.719
Frequency of fruit consumption						
Very rarely or not at all	1.328	(0.628–2.809)	0.458	1.425	(0.640–3.175)	0.386
One	1			1		
Two	0.925	(0.536–1.596)	0.779	1.473	(0.821–2.641)	0.194
Three	0.741	(0.328–1.675)	0.471	2.125	(0.925–4.879)	0.076
More than three	0.421	(0.171–1.038)	0.060	1.602	(0.667–3.849)	0.292
Frequency of meat consumption						
Very rarely or not at all	4.518	(0.748–27.290)	0.100	6.578	(1.105–39.165)	0.039
2–3 times a month	1.369	(0.322–5.812)	0.670	6.857	(1.799–26.126)	0.005
Once a week	0.917	(0.425–1.981)	0.826	1.246	(0.559–2.777)	0.591
2–3 times a week	0.993	(0.609–1.618)	0.977	0.903	(0.537–1.518)	0.701
Daily	1			1		
Frequency of carbonated or sweetened drink consumption						
Very rarely or not at all	0.185	(0.064–0.532)	0.002	0.116	(0.040–0.334)	<0.001
2–3 times a month	0.372	(0.122–1.131)	0.081	0.089	(0.027–0.289)	<0.001
Once a week	0.255	(0.088–0.736)	0.012	0.147	(0.051–0.426)	<0.001
2–3 times a week	0.555	(0.192–1.605)	0.277	0.323	(0.113–0.929)	0.036
Daily, more than one serving	0.423	(0.129–1.392)	0.157	0.303	(0.092–0.994)	0.049
Daily, one serving	1			1		
Frequency of fresh juice or smoothie consumption						
Very rarely or not at all	0.458	(0.124–1.685)	0.240	1.305	(0.306–5.560)	0.719
2–3 times a month	0.689	(0.185–2.567	0.579	1.447	(0.334–6.265)	0.621
Once a week	0.755	(0.195–2.924)	0.684	0.799	(0.174–3.664)	0.772
2–3 times a week	1.120	(0.277–4.529)	0.873	1.190	(0.249–5.692)	0.828
Daily, one serving	1			1		
Frequency of fish or seafood consumption						
Very rarely or not at all	1.602	(0.650–3.950)	0.306	2.013	(0.775–5.232)	0.151
2–3 times a month	1.218	(0.513–2.892)	0.655	1.871	(0.751–4.661)	0.179
Once a week	1			1		
2–3 times a week	2.118	(0.872–5.147)	0.097	1.738	(0.678–4.458)	0.250
Frequency of sweet/pastry consumption						
Very rarely or not at all	1.827	(0.708–4.710)	0.213	1.391	(0.510–3.792)	0.519
2–3 times a month	3.392	(1.378–8.354)	0.008	2.845	(1.055–7.677)	0.039
Once a week	1.152	(0.585–2.267)	0.682	1.172	(0560–2.454)	0674
2–3 times a week	1.409	(0.819–2.424)	0.216	2.080	(1.180–3.667)	0.011
Daily	1			1		
Frequency of pasta, rice, or cereal consumption						
Very rarely or not at all	3.200	(0.942–10.876)	0.062	0.885	(0.251–3.128)	0.850
2–3 times a month	3.872	(1.462–10.254)	0.006	1.382	(0.536–3.561)	0.503
Once a week	1.841	(0.768–4.414)	0.172	0.604	(0.260–1.402)	0.241
2–3 times a week	1.817	(0.800–4.130)	0.154	0.807	(0.377–1.729)	0.807
Daily	1			1		
Daily bread consumption						
More than 12 slices	1			1		
8–12 slices	1.811	(0.133–24.555)	0.655	2.727	(0.175–42.469)	0.474
5–7 slices	0.315	(0.032–3.115)	0.323	0.597	(0.053–6.762)	0.677
1–4 slices	0.249	(0.026–2.349)	0.225	0.514	(0.048–5.551)	0.583
Very rarely or not at all	0.514	(0.052–5.035)	0.567	0.801	(0.071–9.059)	0.858
Frequency of fast-food consumption						
Very rarely or not at all	0.643	(0.112–3.689)	0.620	1.174	(0.190–7.242)	0.862
2–3 times a month	0.323	(0.058–1.788)	0.196	0.482	(0.081–2.853)	0.421
Once a week	0.272	(0.050–1.496)	0.134	0.364	(0.062–2.141)	0.264
2–3 times a week	0.121	(0.021–0.691)	0.018	0.224	(0.036–1.380)	0.107
Daily	1					
Frequency of dairy consumption						
Very rarely or not at all	0.861	(0.308–2.403)	0.774	0.801	(0.269–2.384)	0.690
2–3 times a month	0.803	(0.326–1.977)	0.633	0.505	(0.182–1.402)	0.190
Once a week	1.935	(0.935–4.007)	0.025	1.796	(0.833–3.873)	0.136
2–3 times a week	1.052	(0.622–1.779)	0.851	1.308	(0.760–2.252)	0.332
Daily	1			1		
Frequency of weekly egg consumption						
Very rarely or not at all	0.316	(0.053–1.891)	0.207	0.525	(0.076–3.617)	0.513
1–2 eggs	0.304	(0.054–1.706)	0.176	0.544	(0.085–3.494)	0.521
3–4 eggs	0.169	(0.030–0.951)	0.044	0.331	(0.051–2.136)	0.245
5–7 eggs	0.298	(0.048–1.829)	0.191	0.563	(0.079–4.002)	0.566
More than 7 eggs	1			1		
Frequency of water consumption per day						
Less than 1 L	1			1		
1 L	1.026	(0.439–2.402)	0.952	2.755	(1.039–7.300)	0.042
2 L	1.574	(0.683–3.629)	0.287	3.015	(1.148–7.300)	0.025
3 L	3.732	(1.277–10.907)	0.016	7.146	(2.176–23.468)	0.001
More than 3 L	2.353	(0.672–8.242)	0.181	2.076	(0.487–8.853)	0.323

Reference category: primary school.

**Table 3:** Multinomial logistic regression analysis of dietary patterns and eating behaviors associated with educational level (high school and middle school vs. primary school).

Independent Variables	High School			Middle School		
	OR	95% CI	*p*-Value	OR	95% CI	*p*-Value
Type of food most often consumed						
Fast-food products	0.546	(0.116–2.562)	0.443	3.712	(0.581–23.715)	0.166
Pizza, snacks, pastries, and sweets	0.457	(0.125–1.674)	0.237	1.860	(0.354–9.772)	0.464
Processed meats and canned products	1			1		
Restaurant-cooked meals	0.265	(0.063–1.110)	0.069	1.040	(0.168–6.433)	0.967
Home-cooked meals	0.211	(0.067–0.665)	0.008	1.159	(0.250–5.370)	0.851
Type of cooked food most often consumed						
Fried food	0.799	(0.330–1.934)	0.618	0.947	(0.397–2.259)	0.902
Wood-/charcoal-grilled food	1.577	(0.445–5.582)	0.480	0.710	(0.177–2.845)	0.628
Grilled food	1.175	(0.466–2.958)	0.733	0.621	(0.240–1.609)	0.327
Oven-cooked food	0.711	(0.326–1.552)	0.392	0.449	(0.205–0.981)	0.045
Boiled or steamed food	0.507	(0.214–1.203)	0.123	0.547	(0.234–1.276)	0.163
Other	1			1		
Type of food products in daily diet						
Vegetables and fruits	0.945	(0.418–2.135)	0.891	0.577	(0.259–1.282)	0.177
Cereals and pasta	0.752	(0.296–1.911)	0.549	0.577	(0.231–1.445)	0.240
Dairy products	1			1		
Fish and seafood dishes	1.656	(0.359–7.632)	0.517	0.352	(0.051–2.415)	0.288
Meat	1.024	(0.463–2.267)	0.953	0.619	(0.285–1.346)	0.227
Eggs	0.341	(0.073–1.592)	0.171	0.435	(0.108–1.758)	0.243
Meat-based products (cold cuts, minced meat, canned food, etc.)	2.138	(0.762–6.000)	0.149	1.153	(0.408–3.259)	0.788
Pizza, sweets, and pastries	1.187	(0.227–6.215)	0.839	1.047	(0.214–5.118)	0.955
Foods rich in fats (lard, bacon, fatty meat, etc.)	1.007	(0.135–7.519)	0.994	0.402	(0.042–3.881)	0.431
Fast-food products (burgers, shawarma, chicken nuggets, French fries, etc.)	1.723	(0.448–6.626)	0.429	0.796	(0.204–3.101)	0.742
Assessment of the amount of food consumed daily						
Chaotic, excessive	2.225	(1.079–4.587)	0.030	2.291	(1.075–4.884)	0.032
Chaotic, insufficient	1.517	(0.833–2.763)	0.173	1.693	(0.910–3.147)	0.096
Moderate, without excess	1			1		
According to the body’s needs, monitoring one’s weight	1.396	(0.807–2.413)	0.233	1.935	(1.105–3.386)	0.021
According to a plan set by a specialist	0.627	(0.170–2.303)	0.481	1.753	(0.525–5.849)	0.361
Assessing receptivity to new foods introduced into their diet						
Generally accepts them easily and is receptive to new foods	1			1		
Generally finds them difficult to accept and is reluctant to try new foods	0.200	(0.120–0.334)	<0.001	0.370	(0.223–0.615)	<0.001
Difficult to assess	0.672	(0.373–1.208)	0.184	0.865	(0.470–1.592)	0.641
How they eat their meals						
Generally eats in a hurry	0.805	(0.451–1.435)	0.462	0.764	(0.420–1.391)	0.379
Often does other activities during meals	0.655	(0.403–1.065)	0.088	0.612	(0.368–1.018)	0.059
Eats calmly and without rushing	1			1		
How they eat their meals at home						
Alone	1			1		
With siblings or friends	1.049	(0.477–2.309)	0.905	1.326	(0.577–3.047)	0.506
With siblings, possibly friends, and parents	0.438	(0.278–0.689)	<0.001	0.587	(0.364–0.946)	0.029

Reference category: primary school.

**Table 4:** Multinomial logistic regression analysis of psychological and behavioral factors associated with educational level (high school and middle school vs. primary school).

Independent Variables	High School			Middle School		
	OR	95% CI	*p*-Value	OR	95% CI	*p*-Value
Evaluation of the child’s immune system						
I believe they have a strong immune system	1			1		
I believe they have a weakened or unbalanced immune system	1.999	(0.893–4.475)	0.092	1.706	(0.748–3.891)	0.204
Periodically uses methods to strengthen the immune system	0.675	(0.371–1.230)	0.199	0.638	(0.338–1.205)	0.166
Experiencing periods of irritability						
Never	1.293	(0.671–2.194)	0.443	1.595	(0.826–3.081)	0.164
Sometimes	1			1		
Frequently	0.808	(0.418–1.560)	0.525	0.879	(0.447–1.729)	0.709
Almost always	0.465	(0.156–1.382)	0.168	0.742	(0.247–2.229)	0.595
Suffering from fatigue						
Never	0.490	(0.250–0.959)	0.037	0.275	(0.132–0.576)	0.275
Sometimes	1			1		
Frequently	2.330	(1.197–4.536)	0.013	2.131	(1.069–4.246)	0.032
Almost always	14.592	(3.563–59.769)	<0.001	8.735	(2.063–36.986)	0.003
Suffering from anxiety or panic attacks						
Never	0.749	(0.418–3.110)	0.797	1.101	(0.608–1.993)	0.752
Sometimes	1			1		
Frequently	1.141	(0.418–3.110)	0.797	1.008	(0.356–2.855)	0.988
Almost always	1.234	(0.224–6.799)	0.809	0.266	(0.030–2.329)	0.266
Experiencing stress						
Never	0.640	(0.339–1.207)	0.168	0.793	(0.420–1.499)	0.476
Sometimes	1			1		
Frequently	2.574	(1.245–5.319)	0.011	1.101	(0.496–2.442)	0.814
Almost always	1.478	(0.488–4.474)	0.489	0.839	(0.267–2.639)	0.764
Experiencing concentration issues						
Never	1.046	(0.632–1.732)	0.862	0.885	(0.526–1.487)	0.644
Sometimes	1			1		
Frequently	1.400	(0.510–3.844)	0.514	1.750	(0.641–4.777)	0.274
Almost always	0.637	(0.180–2.259)	0.485	1.249	(0.370–4.221)	0.720
Dealing with insomnia						
Never	1.282	(0.758–2.168)	0.354	1.064	(0.624–1.814)	0.818
Sometimes	1			1		
Frequently	1.055	(0.347–3.203)	0.925	0.694	(0.212–2.273)	0.546
Almost always	0.776	(0.158–3.821)	0.755	0.339	(0.057–2.025)	0.235
Experiencing depressive states						
Never	1.243	(0.654–2.361)	0.507	1.059	(0.540–2.075)	0.868
Sometimes	1			1		
Frequently	0.121	(0.027–0.535)	0.005	0.527	(0.129–2.154)	0.373
Almost always	0.160	(0.027–0.958)	0.045	0.142	(0.020–1.026)	0.053
Sociability and communication						
Yes	1.896	(0.881–4.083)	0.102	1.023	(0.482–2.169)	0.954
No	1			1		
Conflict with peers						
Yes	2.752	(1.136–6.670)	0.025	3.428	(1.418–8.290)	0.006
No	1			1		
Practicing exercise/sports						
No	1			1		
Yes, very rarely	0.965	(0.394–2.363)	0.938	0.634	(0.247–1.626)	0.343
Yes, 2–3 times a week	0.482	(0.200–1.158)	0.103	0.561	(0.226–1.391)	0.212
Yes, daily for under an hour	0.231	(0.078–0.684)	0.008	0.487	(0.168–1.410)	0.184
Yes, daily for at least an hour	0.590	(0.230–1.512)	0.272	0.915	(0.349–2.400)	0.856
Number of hours spent in front of the TV, tablet, computer, or phone						
Daily, a maximum of 1 h	0.245	(0.093–0.645)	0.004	0.195	(0.073–0.519)	0.001
Daily, 2–3 h	0.324	(0.144–0.727)	0.006	0.261	(0.114–0.597)	0.001
Daily, 4–5 h	0.415	(0.180–0.954)	0.038	0.388	(0.166–0.907)	0.029
Daily, 6–7 h	0.613	(0.237–1.585)	0.313	0.714	(0.276–1.850)	0.488
Daily, more than 8 h	1			1		
A few hours 2–3 times a week	0.116	(0.026–0.512)	0.004	0.108	(0.022–0.534)	0.006
Very rarely has access to these devices	0.423	(0.073–2.435)	0.335	0.304	(0.045–2.066)	0.223
Does not have access to these devices	0.617	(0.064–5.915)	0.675	0.278	(0.019–4.019)	0.347

Reference category: primary school.

## Data Availability

The original contributions presented in this study are included in this article; further inquiries can be directed to the corresponding author.
